# miR-200c Sensitizes Breast Cancer Cells to Doxorubicin Treatment by Decreasing TrkB and Bmi1 Expression

**DOI:** 10.1371/journal.pone.0050469

**Published:** 2012-11-29

**Authors:** Florian Kopp, Prajakta S. Oak, Ernst Wagner, Andreas Roidl

**Affiliations:** Pharmaceutical Biotechnology, Department of Pharmacy, Ludwig-Maximilians-Universität München, Munich, Germany; King Faisal Specialist Hospital & Research center, Saudi Arabia

## Abstract

Acquired resistance to classical chemotherapeutics is a major obstacle in cancer treatment. Doxorubicin is frequently used in breast cancer therapy either as single-agent or in combination with other drugs like docetaxel and cyclophosphamide. All these chemotherapies have in common that they are administered sequentially and often result in chemoresistance. Here, we mimicked this pulse therapy of breast cancer patients in an *in vitro* cell culture model, where the epithelial breast cancer cell line BT474 was sequentially treated with doxorubicin for several treatment cycles. In consequence, we obtained chemoresistant cells displaying a mesenchymal-like phenotype with decreased levels of miR-200c. To investigate the involvement of miR-200c in resistance formation, we inhibited and overexpressed miR-200c in different cell lines. Thereby, the cells were rendered more resistant or susceptible to doxorubicin treatment. Moreover, the receptor tyrosine kinase TrkB and the transcriptional repressor Bmi1 were identified as miR-200c targets mediating the drug resistance. Hence, we provide a mechanism of acquired resistance to doxorubicin that is caused by the loss of miR-200c. Along with this, our study demonstrates the complex network of microRNA mediated chemoresistance highlighting the challenges in cancer therapy and the importance of novel microRNA-modulating anticancer agents.

## Introduction

Breast cancer is the most common malignancy in women with 230000 estimated new cases and 40000 estimated deaths in the United States in 2012 [1]. Even though early detection methods and treatment options greatly improved due to a better understanding of the underlying molecular mechanisms, resistance to classical chemotherapeutics is still a tremendous challenge for breast cancer therapy. About 30% of all breast cancer patients who are successfully treated at early stages are suffering a relapse accompanied by metastasis and chemoresistance to classical drugs [2], [3]. While the response rates for first-line chemotherapy including anthracyclines and taxanes are up to 70%, the response rate falls to only 20 to 30% after disease progression. Besides metastasis, this acquired chemoresistance is a major obstacle in the treatment of breast cancer [4], [5]. Hence, an advancement of the treatment by avoiding drug resistance and a better prediction of chemotherapy efficacy would improve the clinical outcome for breast cancer patients.

microRNAs are endogenous, non-coding RNAs of approximately 22 nucleotides that target various genes either by degrading the mRNA or by repressing the translation [6], [7]. Moreover, microRNAs are shown to be dysregulated in many cancers, such as breast, prostate, colon and lung. Thereby, microRNAs can function as onco-miRs or tumor-suppressor-miRs depending on their respective target genes [8], [9]. Previous studies have also shown that microRNAs are able to modulate the sensitivity of cancer cells to chemotherapeutic drugs and therefore contribute to the acquisition of chemoresistance [10], [11], [12], [13], [14].

miR-200c has been reported to regulate epithelial to mesenchymal transition (EMT) by targeting the transcriptional E-Cadherin repressors Zeb1 and Zeb2 [15], [16], [17]. Thus, high miR-200c levels determine an epithelial phenotype of cancer cells which is defined by an elevated E-Cadherin expression, a low migratory capacity and a cobble-stone-like cell morphology [18], [19], [20]. Recent findings suggest that loss of miR-200c may regulate resistance to chemotherapeutics, such as paclitaxel or cisplatin [21], [22]. However, an exact mechanism of miR-200c dependent acquired chemoresistance had yet to be elucidated.

In this study, we mimicked the sequential doxorubicin treatment of breast cancer in an *in vitro* cell culture system using the epithelial breast cancer cell line BT474. The repeated treatment with doxorubicin resulted in a molecular evolution of the tumor cells accompanied by the acquisition of a mesenchymal-like and chemoresistant phenotype which was characterized by a significant down-regulation of miR-200c. Furthermore, we proved in two different breast cancer cell lines that either inhibition or overexpression of miR-200c was sufficient to increase doxorubicin resistance or susceptibility, respectively. Finally, TrkB and Bmi1 were identified as two miR-200c target genes responsible for the acquisition of chemoresistance. Thus, the study provides new insights into the complex regulation of acquired chemoresistance caused by the down-regulation of miR-200c.

## Materials and Methods

### Primary Antibodies

E-Cadherin (C20820, Transduction Laboratories); Vimentin (V9) (SC-6260, Santa Cruz); TrkB (H-181) (SC-8316, Santa Cruz); Akt (#9272, Cell Signaling); p-Akt (S-473) (#4051, Cell Signaling); Bmi1 (PAI-16973, Thermo Scientific); p53 (DO-1) (SC-126, Santa Cruz), Actin (I-19) (SC-1616, Santa Cruz); α-Tubulin (DM-1A) (T9026, Sigma).

### Cell Culture

The breast cancer cell lines BT474 and MDA-MB 436 were obtained from Cell Line Services (Eppelheim, Germany) and cultivated according to supplier’s instructions. Briefly, BT474 cells were grown in RPMI 1640 medium (Gibco) supplemented with 10% fetal calf serum (FCS) and 2 mM glutamine (Gibco) at 37°C and 5% CO_2_. MDA-MB 436 cells were cultivated in L-15 Leibovitz medium (Biochrom) supplemented with 10% FCS and 2 mM glutamine at 37°C without CO_2_.

### Molecular Evolution Assay

The epithelial breast cancer cell line BT474 was treated with 50 nM doxorubicin (doxorubicin hydrochloride, Sigma) for 72 hours when cells reached a confluency of 80%. After treatment doxorubicin containing medium was replaced by fresh medium. As soon as cells recovered, they were seeded for RNA isolation, cell lysis (protein), cytotoxicity assays and the next treatment cycle. In this manner four rounds of the Molecular Evolution Assay were performed. To obtain the appropriate rate of cell death in the Molecular Evolution Assay, several doxorubicin concentrations were tested in a preliminary experiment. Thereby, 50 nM was obtained as the most suitable concentration.

For cytotoxicity assays 5000 cells were seeded in 96-well plates after each round of the Molecular Evolution Assay. 24 hours after seeding cells were treated with different concentrations of doxorubicin for 72 hours. After incubation a CellTiter Glo assay (Promega) was performed following manufacturer’s instructions.

### Quantitative RT-PCR

For mRNA quantification total RNA was isolated with the miRCURY RNA Isolation Kit (Exiqon) followed by a reverse transcription using Transcriptor High Fidelity cDNA Synthesis Kit (Roche) according to manufacturers’ protocols. Quantitative RT-PCR was performed on a LightCycler 480 system (Roche) using UPL Probes (Roche) and Probes Master (Roche). Genes of interest were normalized to GAPDH as reference using the ΔC_T_ method. The following probes and primer sequences were used: E-Cadherin, UPL Probe #35, left primer: CCCGGGACAACGTTTATTAC, right primer: GCTGGCTCAAGTCAAAGTCC; TrkB, UPL Probe #2, left primer: AGTGCCTCTCGGATCTGGT, right primer: TTTCTGGTTTGCGATGAAAAT; Bmi1, UPL Probe #54, left primer: TGTAAAACGTGTATTGTTCGTTACC, right primer: CAATATCTTGGAGAGTTTTATCTGACC; GAPDH, UPL Probe #60, left primer: AGCCACATCGCTCAGACAC, right primer: GCCCAATACGACCAAATCC.

For microRNA quantification total RNA was isolated with the miRCURY RNA Isolation Kit. cDNA synthesis was carried out by a microRNA specific reverse transcription using Transcriptor High Fidelity cDNA Synthesis Kit and a stem loop primer (SLP) which was specific for only one particular microRNA [23], [24], [25]. Quantitative RT-PCR was then performed on a LightCycler 480 system using SYBR Green I Master (Roche). The expression of miR-200 family members (miR-141, miR-200a, miR-200b, miR-200c, miR-429) was normalized to miR-191 as reference [26] using the ΔC_T_ or ΔΔC_T_ method. All sequences of the primers used for the microRNA quantification are shown in the supplement (Table S1).

### miR-200c Inhibition

BT474 and MDA-MB 436 cells were seeded in 96-well plates at a density of 3000 cells per well. The following day cells were transfected with 50 nM miRCURY LNA microRNA Inhibitor for hsa-miR-200c (miR-200c inhibitor) (Exiqon) or miRCURY LNA microRNA Inhibitor Control Negative Control A (scrambled control) (Exiqon) using Lipofectamine 2000 (Invitrogen) according to manufacturer’s protocol.

24 hours after transfection cells were treated with different concentrations of doxorubicin for 72 hours followed by a CellTiter Glo assay.

### miR-200c Overexpression

MDA-MB 436 and BT474 cells were transfected in 6-well plates with 100 pmol Pre-miR miRNA Precursor of hsa-miR-200c (pre-miR-200c) (Ambion) or Pre-miR miRNA Negative Control (scrambled control) (Ambion) using Lipofectamine 2000 according to manufacturer’s protocol. After three consecutive transfections cells were harvested for RNA isolation, cell lysis (protein) and cytotoxicity assays.

For cytotoxicity experiments transfected cells were plated on 96-well plates at a density of 3000 cells per well. 24 hours after seeding cells were treated with different concentrations of doxorubicin for 72 hours followed by a CellTiter Glo assay.

### Cell Lysis and Immunoblotting

Cells were washed with PBS and incubated at 4°C with lysis buffer (Promega) supplemented with phosphatase and protease inhibitors (Santa Cruz and Roche). Cellular debris was removed by centrifugation. Protein estimation was performed using the Micro BCA Protein Assay Kit (Thermo Scientific). Samples were suspended in SDS sample buffer containing β-mercaptoethanol, boiled for 5 minutes and subjected to SDS-PAGE. For western blot analysis, proteins were transferred to nitrocellulose membranes and incubated with the appropriate antibodies. Signals were developed using either Peroxidase Horse Anti-Rabbit IgG Antibody or Peroxidase Goat Anti-Mouse IgG Antibody (Vector Laboratories) and Lumi-Light^PLUS^ Western Blotting Substrate (Roche). Western blot quantification was carried out using ImageJ software [27].

### Immunofluorescence

Cells were seeded on a cover slip and fixed with 4% paraformaldehyde. Cover slips were blocked with 10% FCS, 1% gelatine and 0.05% Triton X-100 and incubated with indicated primary antibodies. Immunofluorescence was visualized using Donkey anti-Mouse IgG (Cy3) (PA1-29773, Thermo Scientific). Counterstaining was performed using 4′-6-diamidino-2-phenylindole (DAPI) (Sigma). Results were analyzed using Zeiss Laser Scanning Microscope LSM 510 Meta and Axiovert 200 software (Zeiss).

**Figure 1:**
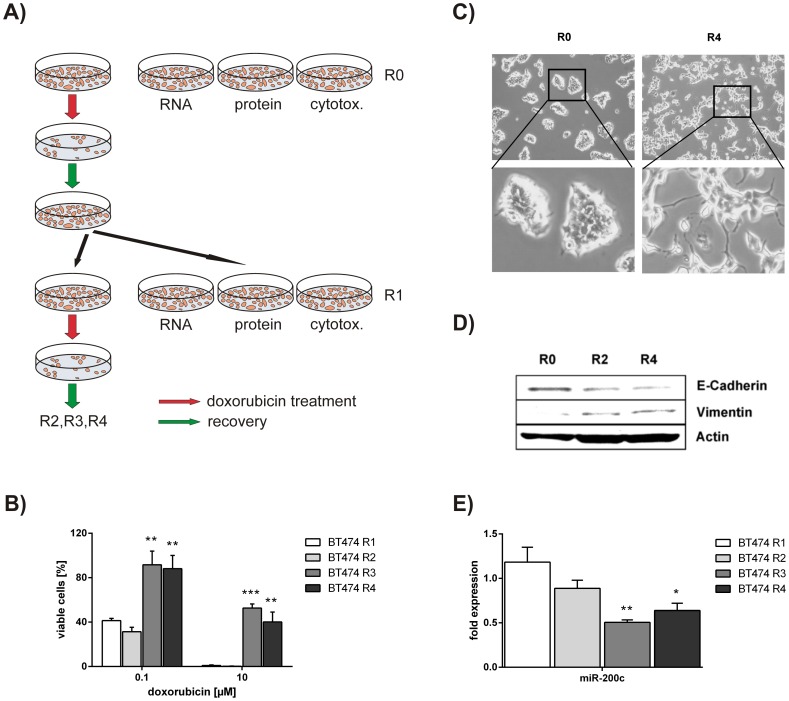
Molecular evolution of breast cancer cells leads to a chemoresistant phenotype and down-regulation of miR-200c. A) Molecular Evolution Assay. The epithelial breast cancer cell line BT474 was sequentially treated with chemotherapy. Cells were treated with 50 nM doxorubicin for 72 hours. Subsequently, medium was replaced by fresh medium until cells recovered and reached a confluency of 80%. Finally, cells were splitted for RNA isolation, cell lysis (protein), cytotoxicity assays and the next treatment round. R0 represents the untreated control cell line, whereas R1, R2, R3 and R4 represents BT474 cells that are treated for one, two, three and four times, respectively. B) Susceptibility to doxorubicin treatment. BT474 cells of R1 to R4 were treated with 0.1 and 10 µM doxorubicin for 72 hours. A CellTiter Glo assay was carried out to determine cell viability. Results are indicated as percentage of viable cells normalized to mock treated cells. C) Cell morphology of untreated and treated BT474 cells. Microscopic pictures (phase contrast) were taken from untreated BT474 cells (R0) and from doxorubicin treated and recovered cells of R4. D) Epithelial and mesenchymal marker expression in BT474 cells of R0, R2 and R4 of the Molecular Evolution Assay. E-Cadherin and Vimentin protein levels were determined by western blot analysis. Actin was used as loading control. E) miR-200c expression in BT474 cells that have undergone molecular evolution. Quantitative RT-PCR was performed to analyze miR-200c levels in BT474 cells of R1 to R4. miR-200c expression was thereby normalized to miR-191. Results are depicted as fold expression compared to the untreated control cell line (R0). Experiments were done in triplicates. For statistical analysis a student’s t-test was performed. (*p<0.05; **p<0.01; ***p<0.001).

**Figure 2:**
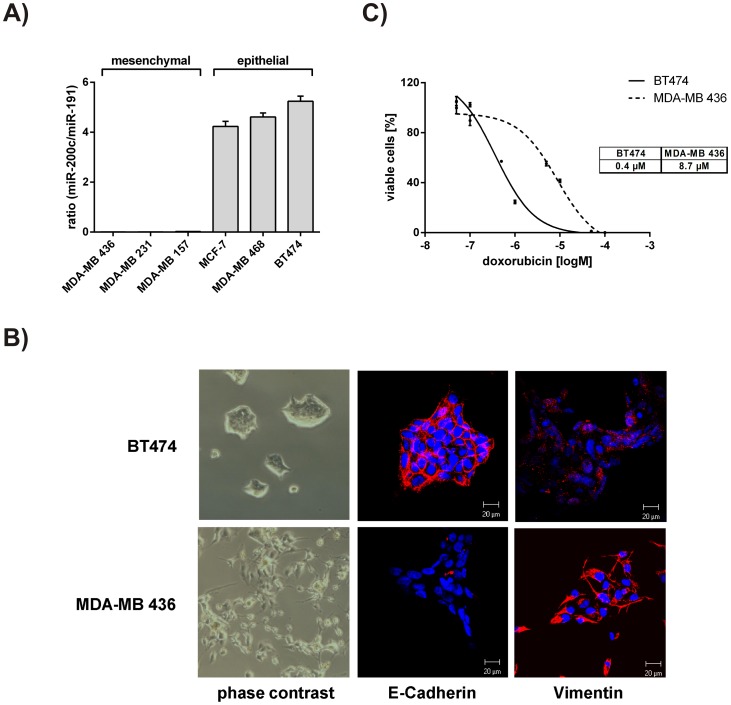
Characterization of the epithelial and the mesenchymal breast cancer cell line BT474 and MDA-MB 436. A) miR-200c levels in a panel of mesenchymal (MDA-MB 436, MDA-MB 231, MDA-MB 157) and epithelial (MCF-7, MDA-MB 468, BT474) breast cancer cell lines. Quantitative RT-PCR was performed to assess the levels of miR-200c. Expression was normalized to miR-191 and presented as ratio. B) Epithelial and mesenchymal phenotype of BT474 and MDA-MB 436 cells. To confirm the different phenotypes, microscopic pictures were taken from both cell lines (phase contrast, left panel). Furthermore, immunofluorescence and laser scanning microscopy was carried out to determine E-Cadherin (red, middle panel) and Vimentin (red, right panel) expression. Nuclei were stained with DAPI (blue). C) Dose reponse curves and IC_50_ values for doxorubicin. BT474 and MDA-MB 436 cells were treated with different concentrations of doxorubicin for 72 hours. Subsequently, a CellTiter Glo assay was performed to determine the percentage of viable cells (normalized to mock treated cells). IC_50_ values are shown in the table. Experiments were done in triplicates. For statistical analysis a student’s t-test was performed. (***p<0.001).

## Results

### Molecular Evolution of Breast Cancer Cells Leads to a Chemoresistant Phenotype and Down-regulation of miR-200c

Occurrence of chemoresistance is one of the major obstacles in chemotherapy of breast cancer patients. Thus, we wanted to mimic the pulse therapy of cancer patients applied in the clinics by using an *in vitro* cell culture system. Therefore, we established the Molecular Evolution Assay, where we treated the epithelial breast cancer cell line BT474 with doxorubicin for several cycles (Figure 1A). After each treatment round cells were harvested for cytotoxicity assays as well as RNA and protein isolation to investigate changes in chemosensitivity and gene expression. The cytotoxicity assays revealed that cells became more resistant from the third treatment cycle on. This effect did not get more significant after the fourth treatment round indicating that the chemoresistant phenotype was obtained within three treatments (Figure 1B). In addition, we could see a change in cell morphology from cobble-stone shape to cells showing slightly more scattering and cytoplasmic processes (Figure 1C). To further characterize the altered phenotype of the doxorubicin treated cells, the protein expressions of E-Cadherin and Vimentin were determined in the rounds of the Molecular Evolution Assay. In accordance with the microscopic pictures, we observed a decrease of E-Cadherin and an increase of Vimentin suggesting an EMT-like mechanism (Figure 1D). As the miR-200 family is regulating EMT by inhibiting transcriptional repressors of E-Cadherin [16], [17], [28], [29], we analyzed the expression levels of all family members (miR-141, miR-200a, miR-200b, miR-200c and miR-429) in BT474 cells. Thereby, miR-200c was the most abundantly expressed family member showing a more than 10-fold higher expression (Figure S1). Hence, we investigated the miR-200c levels during the Molecular Evolution Assay and observed a significant down-regulation within three rounds without any further decrease after the fourth treatment (Figure 1E).

**Figure 3:**
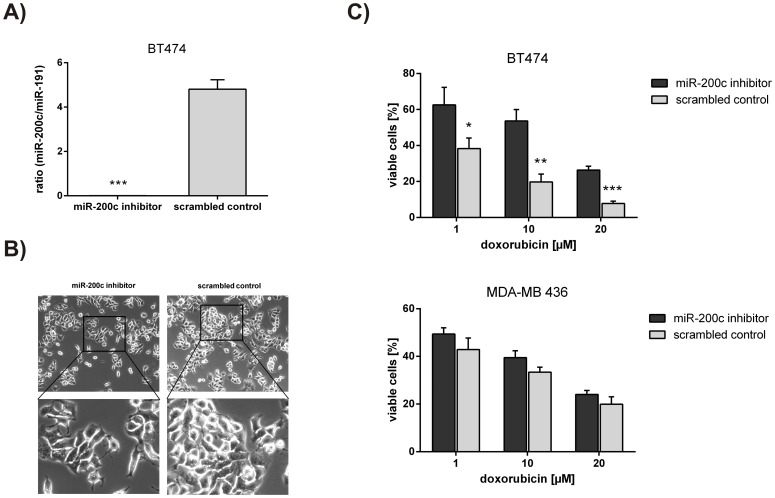
Inhibition of miR-200c in BT474 cells causes chemoresistance to doxorubicin treatment. A) miR-200c expression after inhibiton. 24 hours after transfection with either miR-200c inhibitor or scrambled control, BT474 cells were harvested for RNA isolation and quantitative RT-PCR. miR-200c expression was normalized to miR-191 and presented as ratio. B) Cell morphology. Micrographs (phase contrast) of BT474 cells were taken 24 hours after transfection with either miR-200c inhibitor or scrambled control. C) Susceptibility to doxorubicin treatment. BT474 and MDA-MB 436 cells, transfected with inhibitor or scrambled control, were treated with 1, 10 and 20 µM doxorubicin for 72 hours. Cell viability was analyzed using CellTiter Glo. Experiments were done in triplicates with at least two biological replicates. For statistical analysis a student’s t-test was performed. (ns = not significant; *p<0.05; **p<0.01; ***p<0.001).

### Characterization of the Epithelial and the Mesenchymal Breast Cancer Cell Line BT474 and MDA-MB 436

To investigate the role of miR-200c in chemoresistance, a panel of different breast cancer cell lines was tested for the abundance of miR-200c. The mesenchymal cell lines (MDA-MB 436, MDA-MB 231 and MDA-MB 157) showed no expression of miR-200c, whereas the epithelial cell lines (MCF-7, MDA-MB 468 and BT474) displayed high levels of miR-200c (Figure 2A). The cell line with the highest miR-200c expression (BT474) and one with no miR-200c expression (MDA-MB 436) were further characterized.

**Figure 4:**
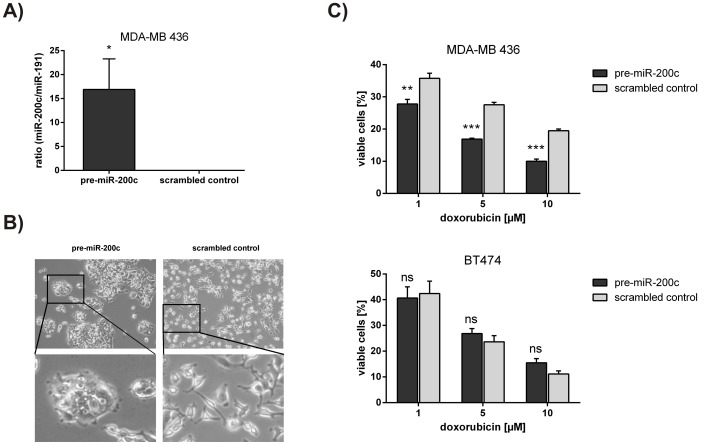
Overexpression of miR-200c in MDA-MB 436 cells increases susceptibility to doxorubicin. A) miR-200c levels after overexpression. After three consecutive transfections with either pre-miR-200c or scrambled control, MDA-MB 436 cells were harvested for RNA isolation and quantitative RT-PCR. miR-200c expression was normalized to miR-191 and presented as ratio. B) Cell morphology. Micrographs (phase contrast) of MDA-MB 436 cells were taken after three consecutive transfections with either pre-miR-200c or scrambled control. C) Susceptibility to doxorubicin treatment. With pre-miR-200c or scrambled control transfected MDA-MB 436 and BT474 cells were treated with 1, 5 and 10 µM doxorubicin for 72 hours. Cell viability was determined by a CellTiter Glo assay. Experiments were done in triplicates with at least two biological replicates. For statistical analysis a student’s t-test was performed. (ns = not significant; *p<0.05; **p<0.01; ***p<0.001).

Consistent with the miR-200c expression, BT474 cells grew in clusters with tight cell-cell junctions representing an epithelial cell type, whereas MDA-MB 436 cells grew as single, spindle-shaped cells resembling a mesenchymal morphology (Figure 2B) [30]. Immunofluorescence confirmed the epithelial phenotype of BT474 in terms of high membrane localized E-Cadherin and low cytoplasmic Vimentin expression. On the other hand, MDA-MB 436 exhibited high Vimentin and only low E-Cadherin levels (Figure 2B) [30]. Finally, we determined the IC_50_ values for doxorubicin in BT474 and MDA-MB 436. Dose response curves revealed that BT474 cells were approximately 22-fold more susceptible to doxorubicin treatment compared to MDA-MB 436 cells (Figure 2C).

Thus, BT474 represents an epithelial breast cancer cell line with high levels of miR-200c and E-Cadherin. On the contrary, MDA-MB 436 displayed a mesenchymal phenotype with no expression of miR-200c, but with a high expression of the mesenchymal marker Vimentin. Along with these findings, BT474 cells were significantly more susceptible to doxorubicin treatment compared to MDA-MB 436 cells. These two cellular systems enabled us to investigate the role of miR-200c in chemoresistance by either inhibiting or overexpressing this particular microRNA.

**Figure 5:**
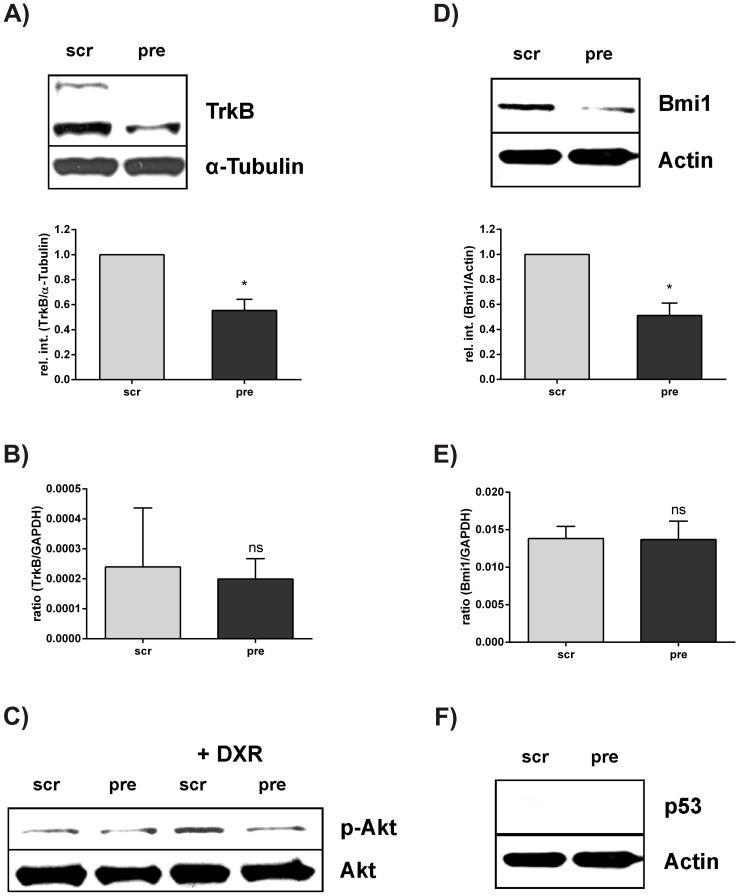
TrkB and Bmi1 protein expression is hampered by overexpression of miR-200c in MDA-MB 436 cells. Total cell lysates of pre-miR-200c (pre) and scrambled control (scr) transfected MDA-MB 436 cells were subjected to western blot analysis and quantitative RT-PCR to determine expression of A) TrkB (gp145 and gp95) protein, B) TrkB mRNA C) p-Akt protein either after treatment with 0.5 µM doxorubicin for 24 hours (right panel) or untreated (left panel), D) Bmi1 protein, E) Bmi1 mRNA and F) p53 protein. α-Tubulin or Actin was used as loading control. Western blot quantification of three independent experiments was carried out by analyzing the relative intensities (rel. int.) of TrkB or Bmi1 normalized to the rel. int. of α-Tubulin or Actin using ImageJ software. For quantitative RT-PCR TrkB and Bmi1 expressions were normalized to GAPDH as reference and presented as ratio. A student’s t-test was performed to assess statistical significance. (ns = not significant; *p<0.05) DXR = doxorubicin.

### Inhibition of miR-200c in BT474 Cells Causes Chemoresistance to Doxorubicin Treatment

We wanted to prove the direct involvement of miR-200c in regulating chemosensitivity. Hence, BT474 and MDA-MB 436 cells were transfected with a miR-200c inhibitor (a locked nucleic acid (LNA) antisense molecule) as well as a scrambled control. As MDA-MB 436 cells have no miR-200c expression, this cell line served as a control to exclude off-target effects of the used LNAs.

By transfecting BT474 cells with the miR-200c inhibitor, we observed significantly reduced levels of miR-200c compared to those cells transfected with the scrambled control (Figure 3A). miR-200c inhibition in BT474 cells also resulted in a more elongated and spindle-shaped cell morphology (Figure 3B). There was no change in cell morphology in MDA-MB 436 cells (data not shown). Most notably, the transfection of BT474 cells with miR-200c inhibitor resulted in significantly more resistant cells, when treated with doxorubicin. As expected, there was no significant effect observable in MDA-MB 436 cells (Figure 3C).

**Figure 6:**
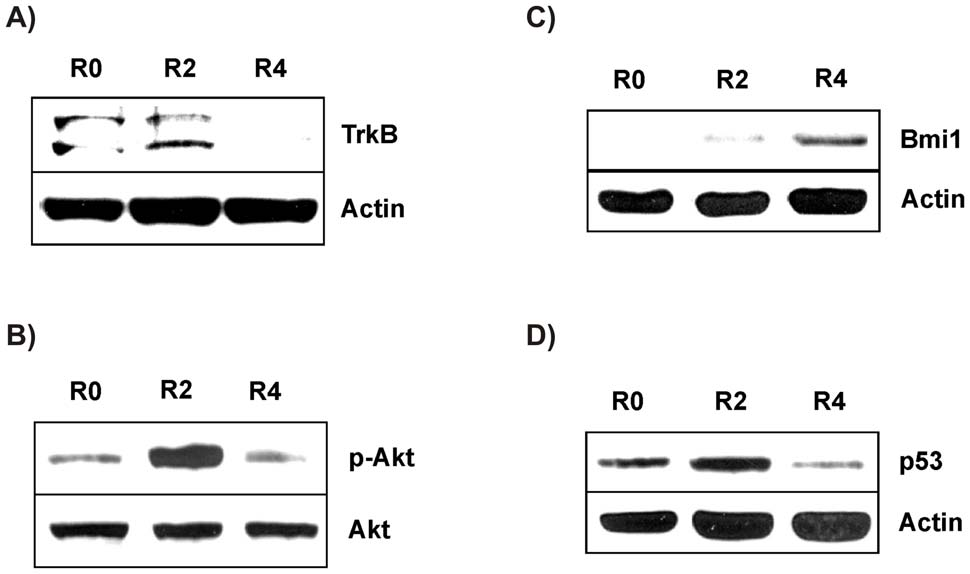
The protein expression of TrkB and Bmi1 is regulated during the rounds of the Molecular Evolution Assay in BT474 cells. Total cell lysates of BT474 cells derived from R0, R2 and R4 of the Molecular Evolution Assay were subjected to western blot analysis to determine protein expression of A) TrkB isoforms, B) p-Akt, C) Bmi1 and D) p53. Actin was used as loading control.

### Overexpression of miR-200c in MDA-MB 436 Cells Increases Susceptibility to Doxorubicin

As demonstrated, miR-200c inhibition in BT474 cells resulted in chemoresistance to doxorubicin. Hence, we investigated whether it was possible to sensitize the miR-200c negative and doxorubicin resistant cell line MDA-MB 436 to doxorubicin treatment by overexpressing miR-200c. Thus, we transfected MDA-MB 436 cells with a microRNA-200c precursor (pre-miR-200c) and gained a high ectopic overexpression of miR-200c (Figure 4A). In accordance with previous studies [15], [16], [18], [19], cell morphology of pre-miR-200c transfected cells changed to a more epithelial-like phenotype with cells growing in clusters (Figure 4B). Finally, we performed a cytotoxicity assay to investigate whether the miR-200c overexpressing cells were more susceptible to doxorubicin treatment. In all doxorubicin concentrations the miR-200c overexpressing cells displayed a higher sensitivity to the treatment. On the other hand, susceptibility of BT474 cells did not increase by further overexpressing miR-200c (Figure 4C).

**Figure 7:**
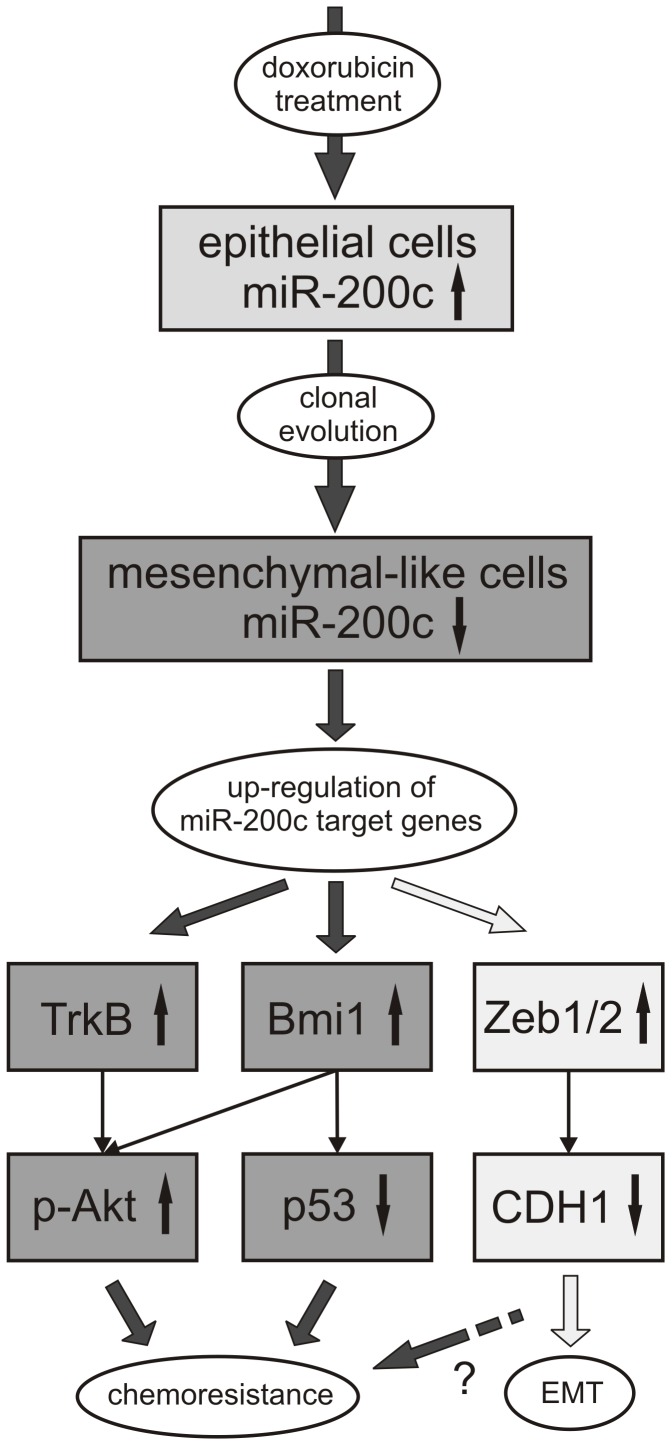
Model of miR-200c regulated chemoresistance. Treatment of epithelial cells expressing miR-200c with doxorubicin leads to clonal evolution of miR-200c low expressing and mesenchymal-like cells. In consequence, a variety of miR-200c target genes are up-regulated. Besides the induction of EMT by the up-regulation of the E-Cadherin (CDH1) repressors Zeb1 and Zeb2 [41], [49], this loss of miR-200c can cause an elevation of resistance factors like TrkB and Bmi1 resulting in enhanced cell survival. This leads to the activation of anti-apoptotic pathways like the phosphorylation of Akt or the degradation of p53, which can be further modulated by a complex crosstalk.

### TrkB and Bmi1 Protein Expression is Hampered by Overexpression of miR-200c in MDA-MB 436 Cells

microRNAs regulate gene expression endogenously either by degrading mRNA or mainly by inhibiting the translational process. Therefore, biological functions and physiological effects of microRNAs can be directly attributed to the silencing of their particular target genes. Thus, we searched for candidates that are targeted by miR-200c and can interfere with cell survival or chemoresistance.

Howe et al. [31] have recently reported that TrkB (NTRK2), a member of the neurotrophic tyrosine receptor kinase family, is a direct target of miR-200c and responsible for anoikis resistance in breast cancer. It has also been shown that in neurons TrkB is involved in differentiation, proliferation and particularly in survival [32].

To clarify if TrkB can be silenced in the cell line MDA-MB 436 upon miR-200c overexpression, we transfected MDA-MB 436 cells with pre-miR-200c or scrambled control. As expected, the protein expression of two TrkB isoforms (gp145 and gp95) was decreased in pre-miR-200c transfected cells compared to scrambled control transfected cells (Figure 5A). On the contrary, TrkB mRNA levels were not altered suggesting that TrkB translation was repressed by miR-200c without degradation of the mRNA (Figure 5B). The receptor tyrosine kinase TrkB is signaling amongst other pathways via PI3K and Akt [32], which plays a pivotal role in cell survival. Thus, we analyzed the phosphorylation status of Akt. However, there were no detectable differences in the p-Akt levels of pre-miR-200c and scrambled control transfected cells. Hence, we treated pre-miR-200c as well as scrambled control transfected MDA-MB 436 cells with doxorubicin for 24 hours to induce Akt dependent survival signaling. Here, we observed enhanced phosphorylation of Akt in the scrambled control transfected cells, whereas miR-200c overexpressing cells still showed low levels of p-Akt (Figure 5C).

Another target of miR-200c is Bmi1, a regulator of stem cell self-renewal and senescence [33]. It has been reported that Bmi1 confers cisplatin and docetaxel resistance in osteosarcoma and prostate cancer, respectively [34], [35]. Thus, we transfected either pre-miR-200c or scrambled control into the mesenchymal cell line MDA-MB 436. As hypothesized, Bmi1 was silenced in the pre-miR-200c transfected cells compared to the scrambled control transfected cells (Figure 5D). Consistent with TrkB, Bmi1 mRNA was also not degraded by miR-200c (Figure 5E). Bmi1 functions as transcriptional repressor leading to the repression of a variety of genes including p16^Ink4a^ and p19^Arf^ of the Ink4a locus. Since repression of p19^Arf^ results in the degradation of p53 by MDM2 leading to anti-apoptotic effects [36], we determined p53 protein expression in pre-miR-200c transfected cells. However, no regulation of p53 protein was observed in MDA-MB 436 as this cell line lacks intrinsic p53 (Figure 5F).

### The Protein Expression of TrkB and Bmi1 is Regulated during the Rounds of the Molecular Evolution Assay in BT474 cells

After demonstrating the effects of miR-200c on the target genes TrkB and Bmi1, we analyzed whether these proteins are also regulated in the Molecular Evolution Assay.

BT474 cells showed a slight elevation of TrkB in the second round (R2) followed by a down-regulation in the last round (R4) (Figure 6A). Moreover, we determined the phosphorylation status of Akt, a downstream target of TrkB and an important regulator of cell survival. Consistent with the regulation of TrkB expression, p-Akt was considerably increased in round 2 (R2) and decreased in round 4 (R4) (Figure 6B).

We also analyzed Bmi1 levels in the protein lysates of the Molecular Evolution Assay of BT474 cells. An up-regulation of Bmi1 was observed with no protein expression in the control round (R0), a slight expression in the second (R2) and a high expression in the fourth round (R4) (Figure 6C). Consequently, we analyzed protein expression of p53 and observed a decline of p53 in round 4 (R4) (Figure 6D). We showed that the Molecular Evolution Assay of BT474 cells led to a regulation of TrkB and Bmi1 protein expression. The respective downstream targets, p-Akt and p53, were modulated accordingly conferring the observed chemoresistance.

## Discussion

In breast cancer treatment classical chemotherapy is largely used besides hormone therapy and novel targeted therapy approaches [4], [37]. For instance, the anthracycline doxorubicin (Adriamycin®) is commonly used as single-agent or in combination with other drugs like docetaxel (Taxotere®) and cyclophosphamide (TAC regimen) in an adjuvant or neo-adjuvant setting [38]. Generally, chemotherapy regimens are administered sequentially, i.e. in case of the TAC regimen patients are treated six times every three weeks [39], [40]. In our study, we mimicked this pulse chemotherapy of breast cancer in an *in vitro* cell culture model by treating the epithelial breast cancer cell line BT474 sequentially in four cycles with doxorubicin. The treatment of BT474 cells with a recovery phase followed by the next treatment cycle resembles the therapy regimen in patients and thereby provides a model for acquired chemoresistance. Indeed, we demonstrated for the first time that Molecular Evolution Assay of BT474 resulted in significantly more resistant cells within three treatment cycles. Acquisition of chemoresistance was accompanied by a change in cell morphology and in the expression of the epithelial marker E-Cadherin and the mesenchymal marker Vimentin suggesting that an EMT-like mechanism might be involved. Besides the well investigated and established regulation of EMT [9], [15], [16], [41], recent findings also suggest that the loss of miR-200c regulates resistance to paclitaxel or cisplatin [18], [21], [22]. However, an exact mechanism of miR-200c dependent acquired chemoresistance had to be elucidated. Here, we show that the sequential doxorubicin treatment of the epithelial breast cancer cell line BT474 leads to an altered phenotype with decreased miR-200c levels resulting in doxorubicin resistance.

To further investigate the role of miR-200c in acquired chemoresistance, we utilized two different cell lines, where we were able to modulate chemoresistance by molecular manipulation of the respective miR-200c levels. First, we inhibited the miR-200c in the epithelial, doxorubicin sensitive and miR-200c highly expressing cell line BT474. As expected, inhibition of miR-200c in BT474 resulted in cells that were significantly more resistant to doxorubicin treatment. Second, we overexpressed miR-200c in the mesenchymal, doxorubicin resistant and miR-200c negative cell line MDA-MB 436. Accordingly, miR-200c expressing MDA-MB 436 became significantly more susceptible to doxorubicin treatment. Thus, we demonstrated in two different cell types that miR-200c alone was able to regulate sensitivity to doxorubicin confirming our findings of the Molecular Evolution Assay.

Since microRNAs are endogenously modulating gene expression either by degrading mRNA or by inhibiting translation, biological functions of these tiny RNAs can be attributed to their targets [6], [7], [8], [9], [42]. Howe et al. [31] have reported that TrkB is targeted by miR-200c in breast cancer conferring anoikis resistance. Moreover, Trk receptors including TrkB are essential for the development and functioning of the nervous system by mediating cell migration, proliferation and most notably survival. Thereby, TrkB is signaling via PLCγ, the MAP kinases or PI3K and Akt [32]. Phosphorylation of Akt plays a pivotal role in cell survival and anti-apoptotic signaling by phosphorylating and thereby inhibiting pro-apoptotic factors like Bad or Caspase 9 [43], [44]. The Molecular Evolution Assay of BT474 cells resulted in a differential expression of TrkB over the cycles with a slight increase in the second round followed by a reduction in the fourth round. Consistent with the TrkB regulation, Akt phosphorylation was remarkably enhanced in the second round and reduced again in the fourth round. The discrepancy in the extent of TrkB and p-Akt regulation can be explained by other clonal selection events influencing the Akt survival pathway. As the Molecular Evolution Assay is based on clonal selection processes, a linear regulation of resistance markers, such as TrkB, p-Akt or p53 is not necessarily expected [45]. Dependent on the particular condition of a treated cell, such as genetic background, phase of cell cycle or extent of DNA damage, distinct survival pathways are preferred to circumvent chemotherapy. To ascertain the role of TrkB as miR-200c dependent resistance factor, we overexpressed miR-200c in the mesenchymal and doxorubicin resistant cell line MDA-MB 436. In accordance with the increased susceptibility to doxorubicin, TrkB protein was silenced in miR-200c overexpressing cells. However, there were no differences in p-Akt levels between pre-miR-200c and scrambled control transfected cells. We ascribed this to a missing stimulus that might induce survival signaling in our transfected cells. Therefore, we treated overexpressing as well as control cells with doxorubicin for 24 hours to activate survival signaling. Indeed, phosphorylation of Akt was induced by doxorubicin treatment in the scrambled control transfected cells, whereas in the pre-miR-200c transfected cells it was blocked at basal levels comparable to untreated cells.

Because microRNAs regulate the expression of numerous target genes [1], [7], [9], we assume that the down-regulation of miR-200c promotes chemoresistance by modulating more than one target gene. Shimono et al. [33] have reported that Bmi1, a member of the polycomb group proteins, is targeted by miR-200c linking breast cancer stem cells with normal stem cells. Furthermore, Bmi1 is involved in the maintenance of stemness and in the regulation of senescence [46], [47], [48]. Thereby, Bmi1 functions as transcriptional repressor of a variety of genes including p16^Ink4a^ and p19^Arf^ of the Ink4a locus. Repression of p19^Arf^ results downstream in the degradation of p53 by MDM2 leading to anti-apoptotic effects [36]. Additionally, recent studies have shown that Bmi1 promotes cisplatin and docetaxel resistance in osteosarcoma and prostate cancer, respectively [34], [35]. Thus, we investigated Bmi1 protein expression over the rounds in the Molecular Evolution Assay of BT474 cells and in miR-200c overexpressing MDA-MB 436 cells. Accordingly, we showed a continuous up-regulation of Bmi1 over the rounds in BT474 cells and a clear down-regulation after miR-200c overexpression in MDA-MB 436 cells. Moreover, we demonstrated that p53 levels were decreased after the fourth cycle of doxorubicin treatment which was in line with the increased chemoresistance in the Molecular Evolution Assay confirming our hypothesis. However, the mesenchymal and resistant cell line MDA-MB 436 was p53 negative independent of the miR-200c levels. This indicates that in MDA-MB 436 Bmi1 may mediate resistance via other pathways than p53, for instance via the PI3K/Akt pathway [35], and that p53 might be silenced through epigenetics, e.g. through promoter methylation. Additionally, miR-200c is a key modulator of a variety of genes which can potentially contribute to chemoresistance. Therefore, we are aware of the possibility that more than these two target genes described in this study can provide different resistance mechanisms dependent on the respective genetic background. Nevertheless, our results demonstrate how one microRNA is able to regulate the circuitry of many pathways to render cancer cells susceptible or resistant to chemotherapy.

In conclusion, doxorubicin treatment of a heterogenous cancer cell population leads to clonal evolution and selection of miR-200c low expressing cells and subsequently to an up-regulation of various target genes including EMT inducing repressors like Zeb1 and Zeb2 [41], [49] as well as chemoresistance inducing factors like TrkB or Bmi1 resulting in enhanced survival signaling (Figure 7).

This study is one step forward in the understanding of miR-200c dependent regulation of chemoresistance. Furthermore, our results provide an insight into the complex interaction network of microRNAs and their numerous target genes highlighting the challenges in cancer therapy and supporting strategies utilizing microRNA-modulating anticancer drugs in the future.

## Supporting Information

Figure S1 **Expression of miR-200 family members in BT474 cells.** miR-200 family screen in BT474 cells. Quantitative RT-PCR was performed to analyze the levels of miR-141, miR-200a, miR-200b, miR-200c and miR-429. The expression of the respective microRNA was normalized to miR-191 as reference and depicted as ratio.
(TIF)

Table S1 **Primer sequences for microRNA quantification.**
(DOC)
