# Supplementary figures and images for: miR-200c Sensitizes Breast Cancer Cells to Doxorubicin Treatment by Decreasing TrkB and Bmi1 Expression
Source: PLoS One. 2012 Nov 29;7(11):e50469. doi: 10.1371/journal.pone.0050469 (PMC3510180; doi:10.1371/journal.pone.0050469)

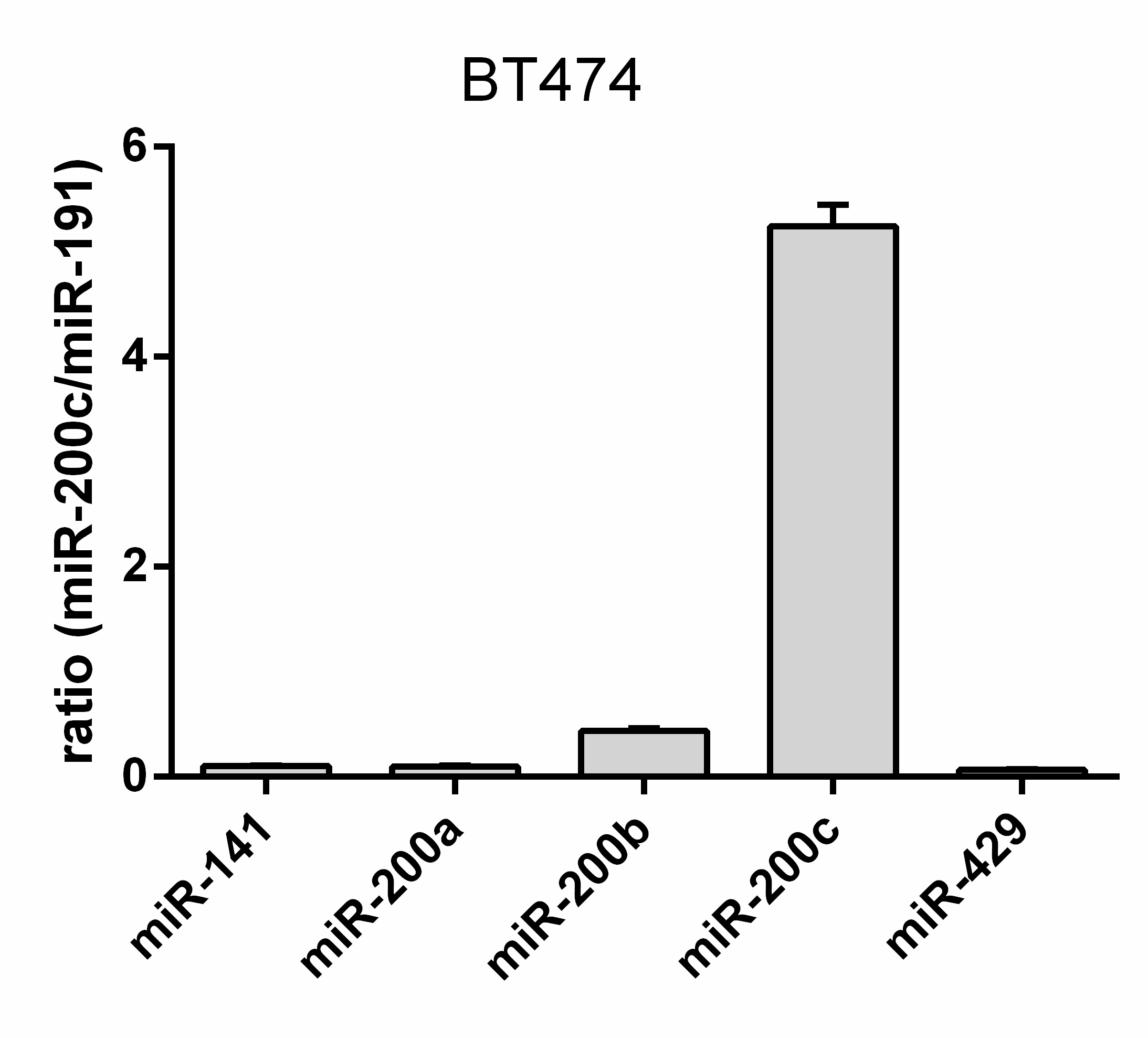

Supplement: Figure S1 — Expression of miR-200 family members in BT474 cells. miR-200 family screen in BT474 cells. Quantitative RT-PCR was performed to analyze the levels of miR-141, miR-200a, miR-200b, miR-200c and miR-429. The expression of the respective microRNA was normalized to miR-191 as reference and depicted as ratio. (TIF) [file pone.0050469.s001.tif]
