# Supplementary material for: miR-200c Sensitizes Breast Cancer Cells to Doxorubicin Treatment by Decreasing TrkB and Bmi1 Expression
Source: PLoS One. 2012 Nov 29;7(11):e50469. doi: 10.1371/journal.pone.0050469 (PMC3510180; doi:10.1371/journal.pone.0050469)
Supplement: Table S1 — Primer sequences for microRNA quantification. (DOC) [file pone.0050469.s002.doc]

| **primer** | **sequence (5’ – 3’)** |
| --- | --- |
| miR-141 SLP | GTTGGCTCTGGTGCAGGGTCCGAGGTATTCGCACCAGAGCCAACCCATCT |
| miR-141 F | GCGTAACACTGTCTGGTAAAGA |
| miR-200a SLP | GTTGGCTCTGGTGCAGGGTCCGAGGTATTCGCACCAGAGCCAACACATCG |
| miR-200a F | GAGTAACACTGTCTGGTAACGA |
| miR-200b SLP | GTTGGCTCTGGTGCAGGGTCCGAGGTATTCGCACCAGAGCCAACTCATCA |
| miR-200b F | GCGTAATACTGCCTGGTAATGA |
| miR-200c SLP | GTTGGCTCTGGTGCAGGGTCCGAGGTATTCGCACCAGAGCCAACTCCATC |
| miR-200c F | GCGTAATACTGCCGGGTAAT |
| miR-429 SLP | GTTGGCTCTGGTGCAGGGTCCGAGGTATTCGCACCAGAGCCAACACGGTT |
| miR-429 F | GAGTAATACTGTCTGGTAAAACC |
| miR-191 SLP | GTTGGCTCTGGTGCAGGGTCCGAGGTATTCGCACCAGAGCCAACCAGCTG |
| miR-191 F | GCGCAACGGAATCCCAAAAG |
| universal R | GTGCAGGGTCCGAGGT |

**Table S1) Primer sequences for microRNA quantification**
